# A barcode database for insects associated with the spread of the Cocoa Swollen Shoot Virus Disease in Côte d’Ivoire

**DOI:** 10.3897/BDJ.13.e144017

**Published:** 2025-03-14

**Authors:** Alain Déron K. Koffi, Régis Babin, Gérard Delvare, Sarah Chérasse, David Ouvrard, Eduardo M. Shimbori, Kouadio Juslin H. Koigny, Serge K. Kpangui, Laure Benoit, Maxime Galan, Christine D.V. Yodé, Mauricette S-W. Ouali N'goran, Julien M. Haran

**Affiliations:** 1 Laboratoire des Milieux Naturels et Conservation de la Biodiversité, Université Félix Houphouët-Boigny, Abidjan, Cote d'Ivoire Laboratoire des Milieux Naturels et Conservation de la Biodiversité, Université Félix Houphouët-Boigny Abidjan Cote d'Ivoire; 2 Centre d’Excellence Africain sur le Changement Climatique, la Biodiversité et l’Agriculture Durable CEA-CCBAD/WASCAL, Abidjan, Cote d'Ivoire Centre d’Excellence Africain sur le Changement Climatique, la Biodiversité et l’Agriculture Durable CEA-CCBAD/WASCAL Abidjan Cote d'Ivoire; 3 CIRAD, UMR PHIM, Abidjan, Cote d'Ivoire CIRAD, UMR PHIM Abidjan Cote d'Ivoire; 4 PHIM Plant Health Institute, Univ Montpellier, CIRAD, INRAE, Institut Agro, IRD, Montpellier, France PHIM Plant Health Institute, Univ Montpellier, CIRAD, INRAE, Institut Agro, IRD Montpellier France; 5 CBGP, CIRAD, INRAE, IRD, InstitutAgro, Univ. Montpellier, Montpellier, France CBGP, CIRAD, INRAE, IRD, InstitutAgro, Univ. Montpellier Montpellier France; 6 ANSES, Laboratoire de la santé des végétaux, Montferrier-sur-Lez, France ANSES, Laboratoire de la santé des végétaux Montferrier-sur-Lez France; 7 Centre de Recherche en Ecologie, Université Nangui Abrogoua, Abidjan, Cote d'Ivoire Centre de Recherche en Ecologie, Université Nangui Abrogoua Abidjan Cote d'Ivoire

**Keywords:** COI barcode, biodiversity, mealybugs, Pseudococcidae, ants, parasitoids, predators, Cocoa swollen shoot virus, *Theobromacacao*, West Africa, Ivory Coast

## Abstract

Swollen Shoot is a viral disease affecting cocoa trees, transmitted by several species of mealybugs (Insecta, Hemiptera, Sternorrhyncha, Pseudococcidae). These insects maintain trophobiotic relationships with a complex and species-rich assemblage of ants protecting them and natural enemies controlling their populations. Here, we provide a curated DNA barcode database to characterise this insect community. Systematic observation of 7,500 cocoa trees was conducted, coupled with the collection of mealybug colonies and associated insect communities (parasitoids, predators and ants). Natural enemies were reared from mealybug colonies collected from 1,430 cocoa trees. Specimens were identified morphologically and sequenced for fragments of the standard DNA barcode region of the COI. We recovered 17 species of mealybugs from the family Pseudococcidae. Amongst these species, eight are new to the Ivorian cocoa orchard: *Dysmicoccusneobrevipes* Beardsley, *Ferrisiadasylirii* (Cockerell), *Maconellicoccusugandae* (Laing), *Paracoccusmarginatus* Williams & Granara de Willink, *Phenacoccussolenopsis* Tinsley, *Planococcusminor* (Maskell), *Pseudococcusconcavocerarii* James and *Pseudococcusocciduus* De Lotto. Three of these species were identified for the first time in cocoa orchards in Africa: *D.neobrevipes*, *Fe.dasylirii* and *Ph.solenopsis*. A total of 54 ant species were identified and represented the first record of these species associated with mealybug colonies in cocoa in Côte d’Ivoire. Amongst the species associated with the mealybugs, 22 primary parasitoids, eight hyperparasitoids, 11 ladybirds beetles (Coccinellidae), seven gall midges (Cecidomyidae), one predatory lepidopteran species and four spider species were identified. Nine species of mealybugs parasitoids are newly recorded in the African cocoa orchards: Acerophagusaff.dysmicocci, *Aloencyrtus* sp., *Anagyruskamali*, Anagyrusaff.pseudococci, *Aenasiusadvena*, Clauseniaaff.corrugata, Gyranusoideaaff.tebygi, Zaplatycerusaff.natalensis (Encyrtidae) and *Coccophaguspulvinariae* (Aphelinidae) and one hyperparasitoid, *Pachyneuronmuscarum* (Pteromalidae). For Côte d’Ivoire in particular, besides the previously mentioned nine parasitoids and one hyperparasitoid, five additional species are recorded for the first time, including four primary parasitoids, *Blepyrusinsularis* (Encyrtidae), *Clauseniacorrugata* (Encyrtidae), *Clausenia* sp. (Encyrtidae), and *Coccidoctonuspseudococci* (Encyrtidae) and one hyperparasitoid, *Cheiloneuruscyanonotus* (Encyrtidae). These results significantly enhance the knowledge of the diversity of the entomofauna associated with Swollen Shoot disease and pave the way for developing control methods based on the natural regulation of its mealybug (Pseudococcidae) vectors.

## Introduction

Cocoa production is crucial to the economies and rural populations of several countries in West and Central Africa. Côte d’Ivoire alone produces over 40% of the world's cocoa, making it the world's leading producer (ICCO 2023). However, production in this country is currently threatened by an expanding lethal disease, the Cocoa Swollen Shoot Virus (CSSV). The virus, from the family Caulimoviridae and genus *Badnavirus* (Muller and Sackey 2005), causes different growth disorders and can be detected through specific symptoms, including red banding on leaf veins, leaf chlorosis, swollen shoots and roots and abnormally small and round pods (Oro et al. 2012, Gyamera et al. 2023). Damage to cocoa leads to a quick decay of farms, with the yield dropping from the first year of infection and, finally, the death of trees within a few years, depending on the virus virulence and cocoa growing conditions (Gyamera et al. 2023). Statistics about the disease outbreaks are scarce in Côte d’Ivoire. A survey conducted at a national scale revealed that 19.5% of prospected cocoa farms (n ≈ 440,000 farms) were CSSV-infected in 2016 (Aka et al. 2020). Today, the percentage of infected farms is probably much higher.

CSSV is transmitted to cacao by mealybugs (Hemiptera, Pseudococcidae), one of the most important families of scale insects (Coccomorpha). The vectoring of CSSV by mealybugs is a non-circulative semi-persistent transmission, which means that the virus is located on the stylets and that a mealybug remains infectious for a relatively short period of two days (Roivainen 1976). At least 64 mealybug species live on the cocoa tree, *Theobromacacao* L. (Malvaceae) in tropical regions, amongst which 22 have been reported in West Africa (García Morales et al. 2016). Half of these species are known to transmit CSSV to *T.cacao* in West Africa (Roivainen 1976). In Côte d’Ivoire, 12 species have been reported on *T.cacao* (García Morales et al. 2016), amongst which seven are known as CSSV vectors (Roivainen 1976), namely *Dysmicoccusbrevipes* (Cockerell), *Ferrisiavirgata* (Cockerell), *Formicococcusnjalensis* (Laing), *Planococcuscitri* (Risso), *Planococcuskenyae* (Le Pelley), *Phenacoccushargreavesi* (Laing) and *Pseudococcuslongispinus* (Targioni Tozzetti). *Fo.njalensis* and *Pl.citri* are by far the most common species on cocoa in Côte d’Ivoire (Obodji et al. 2015, N'Guessan et al. 2019, N’guettia et al. 2021).

Around 70 ant species have been found to interact with CSSV vector mealybugs on cocoa farms in Ghana, most of which belong to genera *Camponotus*, *Crematogaster*, *Oecophylla*, *Pheidole* and *Tetramorium* (Strickland 1951a, Campbell 1975). Tending ants protect them from rain or natural enemies by enclosing them in carton or soil tents. Ants also help mealybugs by consuming the honeydew they produce which helps colonies by limiting fungal growth (Strickland 1951b, Dufour 1991). They may transport mealybugs between their mandibles, but only on short distances (Strickland 1951a). These ants, therefore, contribute to the establishment of large colonies of mealybugs on cocoa trees and possibly to CSSV outbreaks. Depending on the mealybug species, the mealybug-ant associations can vary from facultative to strict. Species with a facultative association can project honeydew away from the colony by contracting the rectum (Way 1963). On the other hand, association with ants is strict for some species due to the quality of the honeydew produced and the inability to project it away from the colony (Way 1963, Jahn and Beardsley 2000, Jahn et al. 2003). *Fo.njalensis*, the most abundant species in West African cocoa plantations, belongs to the latter category. In contrast, *Pl.citri* and other species have a facultative association with the tending ants (Strickland 1951b).

Worldwide, mealybugs have a wide range of natural enemies, including parasitoid wasps and predators (Franco et al. 2009). The highly polyphagous and cosmopolitan *Pl.citri* alone is associated with about 140 chalcidoid species, some of them parasitoids and others hyperparasitoids, mostly belonging to the family Encyrtidae (Hymenoptera, Chalcidoidea), but also Aphelinidae, Eriaporidae, Eulophidae, Pteromalidae and Signiphoridae (Noyes 2019). *Fo.njalensis* has a more restricted distribution in Central and West Africa and is associated with 40 parasitoid species, mostly in the family Encyrtidae (Noyes 2019). Data on parasitoids of cocoa mealybugs are scarce, with many unidentified species, mainly from studies conducted in Ghana (Bigger 2012). Strickland (1951a) reported 13 encyrtid species on *Fo.njalensis*, mainly in the genera *Aenasius* and *Anagyrus*. More recently, Ackonor and Mordjifa (1999) and Ackonor (2002) identified seven species of encyrtids on both *Fo.njalensis* and *Pl.citri* and reported seven other unidentified wasps and a parasitoid fly of the genus *Cryptochetum* (Diptera, Cryptochetidae). For Côte d’Ivoire, Risbec (1949) and Risbec (1955) identified eight species of encyrtids on *Fo.njalensis* and three more species of the families Megaspilidae, Platygastridae and Signiphoridae. Mealybugs are also the prey of various generalist predators, including ladybird beetles (Coleoptera, Coccinellidae), lacewings (Neuroptera, Chrysopidae), gall midges (Diptera, Cecidomyiidae), and various spiders (Strickland 1951a, Dufour 1991, Ackonor 2002).

Effectively managing CSSV requires a thorough knowledge of the insects transmitting the virus to cocoa trees. In addition, it is crucial to identify species capable of naturally regulating vector populations as these could be good candidates for biological control. Identification of these insects, typically based on morphological characteristics, presents challenges, especially for non-specialists (Pacheco da Silva et al. 2014, Puig et al. 2021b). Pseudococcidae are highly diversified and different regions harbour different species, sometimes very similar morphologically, making taxonomic identification difficult and posing a major obstacle to the research and management of these species (Puig et al. 2021b). Species identification of scale insects is almost entirely based on the morphological characteristics found in the adult female; adult males and immature stages can, in most cases, not be identified to species and often renders inconclusive results (Pacheco da Silva et al. 2014). In addition, morphological identification usually requires that the specimens be processed, cleared of their internal contents and slide-mounted which is time-consuming and requires special tools and training. To overcome these identification challenges, the DNA Barcoding approach is a powerful alternative and complementary approach that has been applied successfully to mealybugs and their natural enemies (Park et al. 2011, Abd-Rabou et al. 2012, Pacheco da Silva et al. 2014, Malausa et al. 2016, Puig et al. 2021b, Puig et al. 2021a, Pacheco da Silva et al. 2021, Nurbaya et al. 2022). However, this method relies on the availability of curated barcode databases and cross-validation by taxonomic experts to ensure the consistency of species concepts and molecular data.

If DNA barcode data have already been provided for cocoa mealybugs (Wetten et al. 2015, Obok et al. 2021, Puig et al. 2021b), our study is the first to provide a complete DNA barcode database for the entomofauna, including ants and natural enemies, associated with the CSSV disease in Côte d'Ivoire and, more generally, in West Africa. Based on intensive sampling in cocoa fields in Côte d’Ivoire, barcode sequences for 131 species of mealybugs, ants and natural enemies and 190 unique haplotype sequences are reported. This dataset paves the way for the use of high-throughput metabarcoding approaches to identify species in this complex community.

## Material and methods

### Sample collection

This study was conducted in Côte d’Ivoire, in cocoa plantations of a network implemented within the Cocoa4Future project (see Acknowledgements). The plantation network comprises 15 sites, geographically covering the entire production area of the country. Hence, our study includes the vast majority of environmental, agronomical and historical cocoa growing conditions (Kouassi et al. 2023); (Table 1).

**Figure 1.**
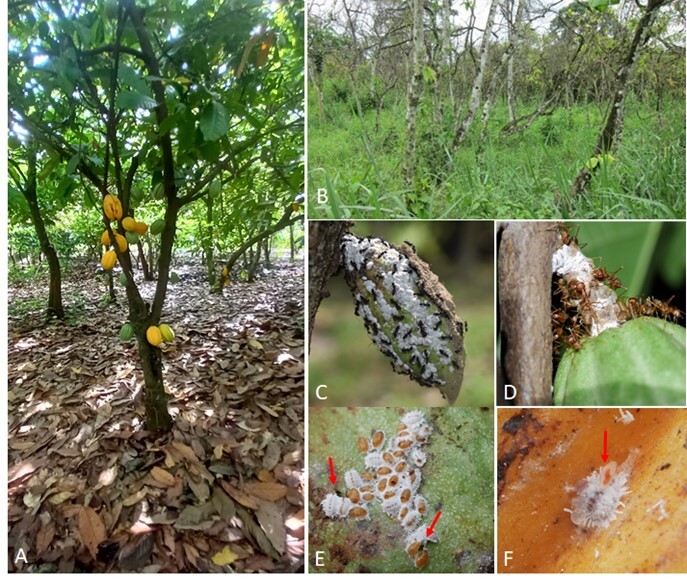
Cocoa plantations in Côte d’Ivoire and mealybug colonies with examples of associated ants and natural enemies. **A** Healthy cocoa plantation; **B** Plantation infected by the cocoa swollen shoot virus; **C** Colony of *Dysmicoccusneobrevipes* associated with ants of the genus *Camponotus*; **D** Colony of *Formicococcusnjalensis* associated with *Oecophyllalonginoda*; **E** Colony of parasitised mealybugs showing mummies and emerged parasitoids (arrows); **F** Cecidomyiid larva feeding on a mealybug (arrow).

Mealybugs and tending ants were sampled in five plantations in each of the 15 sites, i.e. 75 plantations in total (Fig. 1A and B). One hundred cocoa trees were thoroughly searched in each plantation, moving across the plot following a zigzag pattern (Sether et al. 2010) to cover the plantation's structural diversity.

The mealybugs were searched for on cocoa trees from the ground up to a height of 2 metres, focusing on green parts, where mealybug colonies are usually found due to the easy access to the cambium, i.e. suckers, cracks in the bark of trunks, flower cushions, flowers, pods, leaves, buds and shoots on branches. Cocoa canopy was also prospected by collecting branches using a pruning saw. However, this method was not systematically applied to all cocoa trees because it is destructive and not always accepted by famers. Nevertheless, it allowed us to sample 214 mealybug colonies from cocoa canopy. When detected on trees, mealybug colonies (Fig. 1C and D) were recorded and approximately ten individuals were collected from each colony along with the tending ants. Insects were collected using soft forceps and preserved in collection tubes containing 90% ethanol pending their transfer to the entomology laboratory of Centre d’Excellence Africain sur le Changement Climatique, la Biodiversité et l’Agriculture Durable/West African Science Service Centre on Climate Change and Adapted Land Use/Université Félix Houphouët Boigny (CEA-CCBAD/WASCAL/UFHB), Bingerville. A total of 1,650 mealybug colonies were collected, including the tending ants when present. Samples were sorted, identified to morpho-species level and stored in 96% ethanol for further morphological and molecular identification.

Sampling of mealybug natural enemies was conducted in 10 cocoa plantations in 11 out of the 15 sites, i.e. 110 plantations in total. For each plantation, natural enemies were obtained through two consecutive methods. First, three cocoa pods with mealybug colonies that covered at least one-third of the pod surface (Fig. 1C) were carefully collected with secateurs. The pods were stored individually in two-litre plastic bottles cut in half and closed with muslin held in place with a rubber band to allow aeration. Second, ten other mealybug colonies were gently collected from trees using a camel-hair brush and introduced into 4.5 ml (75 x 12 mm) dry collection tubes (without ethanol), closed with muslin. In all, 1,430 mealybug colonies (three infested pods plus ten mealybug colonies in tubes x 110 plantations) were collected. These samples were stored in the laboratory at room temperature and the emergence of the natural enemies was monitored every five days for 20 days. During observation, insects emerging from the tubes and pods were captured, classified into morpho-species and preserved in microtubes containing 96% ethanol. For each mealybug colony, the tending ants were also separately collected in tubes containing 90% alcohol. The species occurrence was assessed from the field and laboratory records for mealybug colonies, tending ants and natural enemies according to the following categories: (-) rare occurrence (= 1-10 occurrences), (+) moderate occurrence (= 10-20 occurrences), (++) regular occurrence (= 20-40 occurrences) and (+++) very frequent occurrence (= 40 and over occurrences).

### Morphological identification

Microscopic preparations were carried out to morphologically identify mealybugs. Specimens were slide-mounted following the protocol established by Streito and Germain (2021). Specimens from which DNA was extracted were also mounted on slides. These specimens followed the same preparation process (Streito and Germain 2021), with the exception of the clearing step using potassium hydroxide (KOH) 10% solution. This step was carried out during extraction, where the soft tissues within the mealybug's body were dissolved by the Proteinase K. Identification keys by Williams (1986), Cox (1989), Williams and Matile-Ferrero (1995), Williams (2004) and Kaydan and Gullan (2012) were used. After using the keys, identifications were confirmed using descriptive papers for species (Hall 1946, De Lotto 1957, Williams 1958, De Lotto 1961, Balachowsky and Matile-Ferrero 1966).

Specimens of ants were dry-mounted and identified using a stereomicroscope. Identification keys from Bolton (1994) and Fisher and Bolton (2016) were used to identify the ants at the genus level. Additional works on the systematics of African ant genera (Bolton 1973, Bolton 1980, Bolton 1982, Bolton 1987), a physical reference collection (Yeo 2006) and online collections on AntWeb (2024) were also used for further identification at the species level.

For parasitoids, specimens were dry-mounted and identified using the CBGP reference collection and identification keys from Compere (1931), Mercet (1931), Compere (1937), Compere (1938), Compere (1939), Bennett (1955), Kerrich (1967), Annecke and Mynhardt (1970), Prinsloo (1988), Noyes and Hayat (1994), Anga and Noyes (1999), Noyes (2000) and Trjapitzin and Triapitsyn (2007) Ladybirds were identified at the genus level using the key by Gourreau (1974). Due to the lack of available expertise, the Cecidomyiidae, Lepidoptera and Araneae were identified at the morphospecies level only.

### DNA extraction, amplification and sequencing

For each morphospecies, one to several specimens were dried at room temperature and placed individually into 96-well microplates for DNA extraction. In total, DNA was extracted from 660 insect specimens. Two negative controls were included in each microplate. DNA extraction from the insect specimens was performed using the EZ-10 96-well plate DNA isolation kit for animals (Biobasic Inc: reference BS437, Canada). All extractions followed the manufacturer’s protocol, including non-destructive lysis by overnight incubation at 55°C with 450 rpm agitation in 300 µl of animal cell lysis solution and 20 µl of proteinase K. For elution, 40 µl of elution buffer was added to each well and left to stand for 5 minutes before a 2-minute centrifugation at 6,000 g. A second elution was performed to recover the total DNA with 40 µl of elution buffer. In total, 80 µl of DNA extract was obtained per well and stored at -20°C in the freezer. After lysis, the individuals were reconditioned in ethanol or mounted as reference "voucher" specimens.

PCR amplification followed the 2-step PCR protocol described by Galan et al. (2018). This method involves two PCR steps, the first being a classical PCR to amplify a portion of the Cytochrome Oxidase I (COI) gene. The DNA of ants and natural enemies was amplified using the universal primers “BB” (Elbrecht et al. (2019); BF3: 5’- CCHGAYATRGCHTTYCCHCG-3’; BR2: 5’- TCDGGRTGNCCRAARAAYCA-3’) in a COI region coding for 418 bp, which are particularly recommended for metabarcoding arthropods of different orders with good taxonomic resolution. In contrast, the DNA of mealybugs was amplified using the specific primer C1J in the COI region coding for 385 bp (Gullan et al. (2003); C1-J-2183: 5’- CAACATTTATTTTGATTTTTTGG-3’; C1-N-2568: 5’- GCWACWACRTAATAKGTATCAT-3’), which enables successful PCR amplification and taxonomic resolution in the Pseudococcidae, a family for which universal COI primers have high amplification failure rates (Malausa et al. 2011). During the second PCR step, i5 and i7 indexes consisting of short 8 bp indexes and P5 and P7 Illumina adapters were added to the 5' end of the DNA sequences to allow the assignment of sequences to each sample after mixing the PCR products for Illumina sequencing. The first PCR step was performed in a final volume of 10 µl, including 5 µl of 2x PCR Multiplex Master Mix (Qiagen, Hilden, Germany), 2.5 µl of ultrapure water, 0.5 µl of each primer (Forward and Reverse at 10 µM) and 1.5 µl of DNA. The PCR conditions were as follows: initial denaturation of DNA at 95°C for 15 minutes, followed by 40 cycles of 30 seconds at 94°C, 45 minutes at 45°C and two minutes at 72°C, with a final extension of 10 minutes at 72°C.

The second PCR was performed in a total volume of 10 µl, including 5 µl of 2x PCR Multiplex kit (Qiagen, Germany), 0.7 µM of each indexed primer and 2 µl of products from the first PCR per sample. The PCR conditions included an initial denaturation at 95°C for 15 minutes, followed by 8 cycles of denaturation at 95°C for 40 seconds, annealing at 55°C for 45 seconds, extension at 72°C for 2 minutes and a final extension step at 72°C for 10 minutes. The PCR products were pooled and sequenced on a MiSeq platform (Illumina).

### Sequence analysis

DNA sequences were sorted using the FROGS pipeline (Escudié et al. 2017) and aligned and manually checked using CodonCode Aligner v. 3.7.1. (CodonCode Corporation, Centerville, MA, USA) to verify the absence of pseudogenes using standard detection methods (Haran et al. 2015). The genetic distances between species were calculated pairwise using the Kimura-2-Parameter (K2P) model (Kimura 1980) in MEGA v.11.0.13 (Tamura et al. 2021), with the "pairwise deletion of gaps" option. Phylogenetic trees were constructed to visualise the observed genetic divergence between species using the Neighbour-joining (NJ) method (Saitou and Nei 1987) with PhyML 3.0 (Guindon et al. 2010). To assess the robustness of the phylogenetic trees, 1000 bootstrap values were generated using PhyML 3.0, providing approximately 50% support for most groups on each branch. The separation of closely-related species is generally based on an intraspecific divergence threshold of 2 - 3% for most insect groups, including Coleoptera, Lepidoptera, Hemiptera and Hymenoptera (Smith et al. 2005, Castro and Dowton 2006, Packer et al. 2009, Bergsten et al. 2012, Mutanen et al. 2012). These thresholds may vary depending on the species groups studied and the geographic distances of sample origins (Bergsten et al. 2012). Therefore, in this study, all species groups with intraspecific distances of ≥ 2% compared to their closest neighbour in the tree were retained and deposited on GenBank (https://www.ncbi.nlm.nih.gov/genbank/). All sequences obtained for the various species groups were compared to the GenBank and/or Bold databases to enable identification and assign names to the sequences.

### Data resources

The sequences obtained for each specimen are deposited in GenBank. A total of 36 sequences from 17 species of Pseudococcidae (Suppl. material 13), 77 sequences from 54 species of Formicidae (Suppl. material 14), 30 sequences from 22 primary parasitoids and nine sequences from seven hyperparasitoids (Suppl. material 15), 15 sequences from Coccinellidae (Suppl. material 16), nine sequences from Cecidomyiidae (Suppl. material 17), four sequences from four species of spiders, one sequence from Lepidoptera and nine sequences from eight parasitoids of predators (Suppl. material 18) have been deposited in GenBank (Tables 2, 3 and 4). All voucher specimens were deposited at CBGP, Montpellier, France, in the CIRAD collection (https://doi.org/10.15454/D6XAKL).

## Results

A total of 305 COI sequences, with coding for a 385 bp fragment, were generated from 17 species of mealybugs (Pseudococcidae) collected from cocoa plantations in Côte d’Ivoire. Pseudococcidae specimens associated with these sequences were identified morphologically to species level (Table 2). The most abundant mealybug species on cocoa were *Formicococcusnjalensis* (Laing), *Planococcuscitri* (Risso) and *Dysmicoccusneobrevipes* Beardsley. Intraspecific distances ranged from 0% to 6.57%, with distances higher than 2% within *Fo.njalensis* (4.57%), *Pl.citri* (2.93%), *Pseudococcuslongispinus* (Targioni Tozzetti) (3.73%) and *Pseudococcusocciduus* De Lotto (6.57%; Table 2). Inter-specific distances were consistent with species concepts based on morphology, with divergences ranging from 4% to 19% (Suppl. materials 1, 2).

For tending ants, 211 COI sequences, coding for a 418 bp fragment, were generated from 54 species collected with mealybug colonies in cocoa plantations. Amongst ant species, 24 were identified at the species level and 30 at the genus level (Table 3). The most abundant ant species tending cocoa mealybugs were *Camponotusacvapimensis* Mayr, *Crematogasterafricana* Mayr, *Lepisiotacocazela* Santschi, *Paratrechinalongicornis* (Latreille), *Pheidolemegacephala* (Fabricius) and two other unidentified species of *Pheidole*. Intraspecific distances ranged from 0% to 6.29%, with distances higher than 2% within *Crematogaster* sp.1 (2.69%), *Ph.megacephala* (2.70%), *Pheidole* sp.1 (3.96%), *Pheidolepunctulata* Mayr (4.98%) and *Pa.longicornis* (6.29%). A large interspecific divergence (ranging from 6.29% to 36%) was observed amongst major neighbouring species groups (Suppl. materials 3, 4).

Natural enemies of mealybugs on cocoa trees include hymenopteran parasitoids, predatory beetles and predatory dipterans (Table 4). In total, 143 COI sequences of the 418 bp fragment were generated from 65 species of natural enemies. The hymenopteran parasitoids include 22 species of primary parasitoids and eight hyperparasitoid species (Table 4). The most abundant species of parasitoids were *Aenasiusabengouroui* (Risbec), *Anagyruskivuensis* Compere, *Coccidoctonuspseudococci* (Risbec), *Leptomastixdactylopii* Howard and one hyperparasitoid, *Cheiloneuruscarinatus* (Compere). Substantial interspecific divergences, ranging from 9.57% to 22.15%, were observed between neighbouring species groups (Suppl. materials 5, 6). Genetic distances also revealed a significant intraspecific variation, ranging up to 8.22%, with higher divergences observed within two primary parasitoids: Anagyrusaff.subproximus (8.22%) and *L.dactylopii* (5.85%). Due to sequencing failure, no COI sequences were obtained for *Gyranusoidea* sp. and *Coccidoxenoides* sp.

The predators of cocoa mealybugs are represented by 11 species of ladybugs beetles (Coccinellidae) spread across four genera, *Hyperaspis*, *Platynaspis*, *Scymnus* and *Nephus* and seven species of gall midges (Diptera), from the family Cecidomyiidae, whose genera and species could not be determined due to the lack of specialists for these groups and the poor condition of specimens after DNA extraction. Although the barcode sequences allowed for the differentiation of seven species of gall midges, these sequences could not be matched to any sequences previously published in online molecular databases. For this group, we thus only used morpho-species concepts. The most abundant predators were two unidentified species of *Nephus* and *Scymnus* and two unidentified species of Cecidomyiidae (Table 4). For coccinellids, all intraspecific variations were ≤ 2%. The morphospecies Cecidomyiidae sp2 shows a higher intraspecific divergence of 2.69%. Interspecific divergences amongst coccinellids and cecidomyiids ranged from 11% to 28% (Suppl. materials 7, 8, 9, 10). Four morpho-species of Lepidoptera from the genera *Niditinea* Petersen (Tineidae), *Spalgis* Moore (Lycaenidae) and *Syringoseca* Meyrick (Oecophoridae) and four morpho-species of spiders from the genera *Psammitis* Menge (Thomisidea), *Myrmarachne* De Geer (Salticidae) and *Theridion* Walckenaer (Theridiidae) were collected from mealybug colonies (Table 4).

Some parasitoids associated with Cecidomyiidae, Coccinellidae and lepidopterans were identified: three species of the genus *Aphanogmus* and one of *Platygaster* (parasitoids of Cecidomyiidae), two species of *Homalotylus* (parasitoids of Coccinellidae) and one species each from the genera *Antrocephalus*, *Apanteles* and *Ooencyrtus* (parasitoids of lepidopterans) (Table 4). No sequence was obtained for *Ooencyrtus* Mayr due to a sequencing failure. Therefore, the species was identified solely based on morphological characteristics (Suppl. materials 11, 12).

## Discussion

This study aimed to explore the diversity of mealybugs as potential vectors of CSSV disease in Côte d’Ivoire, as well as that of their tending ants and natural enemies. The results show that the COI gene fragments enable species-level identification in this taxon-rich community. In all functional groups, however, some species showed significant intraspecific divergences suggesting possible cases of complexes of cryptic or closely-related species. *Ps.occiduus*, for example, with a maximum divergence of 6.57%, indicates the possible presence of cryptic species, as no morphological differences were observed between divergent lineages. In contrast, the 4.57% of divergence between specimens of *Fo.njalensis* was associated with a slight morphological divergence, already reported by Hall (1946) and Padi and den Hollander (1996), supporting the hypothesis that *Fo.njalensis* may be a complex of cryptic species. The problem of delimiting complexes of cryptic species in most cases was resolved using an integration of morphology and molecular information, which was especially true in some genera, such as the parasitic wasp *Anagyrus* and the mealybug *Planococcus*. After analysing their DNA barcodes, we revisited their morphological identification and the careful examination allowed the observation of subtle morphological differences and the attribution of correct species names to all individuals. In the genus *Planococcus*, for instance, the complex *Pl.citri*/*kenyae*/*minor* is formed by three cryptic species (Dufour (1991) observed this for *citri* and *kenyae*) and are easily separated by their barcodes. Detailed morphological examination of voucher specimens revealed useful diagnostic characters, based on criteria defined by Cox (1989) for *Pl.citri*/*minor*. In tending ants and for most parasitoids, the high intraspecific genetic distance could not be correlated to morphological divergence. Slight morphological divergences were observed between individuals bearing distant haplotypes in *Clausenia*, *Zaplatycerus* and *Acerophagus* only. The clarification of the taxonomic status of these lineages is beyond the scope of this study. These cases deserve further morphological investigation as well as the complementary use of longer fragments of COI and nuclear genes to verify the validity of the species concepts used here.

This study expands the previous inventories of cocoa-associated fauna of mealybugs and their tending ants and natural enemies in Côte d’Ivoire and in West Africa in general. Our findings are comparable to those obtained in Togo, where Dufour (1991) recorded ten species of mealybugs on cocoa and in Ghana, where Strickland (1947) and Campbell (1983) reported ten species and Strickland (1951a) identified eight species plus nine unnamed species of mealybug in cocoa. However, mealybug communities significantly differ amongst studies. Seven species, namely *Paraputoanomalus* (Newstead), *Delococcustafoensis* (Strickland), *Paraputoloranthi* (Matile-Ferraro), *Formicococcusceltis* (Strickland), *Tylococcuswestwoodi* Strickland, *Nipaecoccusmasakensis* (James) and *Pseudococcuscalceolariae* (Maskell) were reported from Togo and/or Ghana, but not in the present study. On the other hand, we identified three species previously unreported on cacao in Africa (García Morales et al. 2016): *D.neobrevipes*, *Fe.dasylirii* and *Ph.solenopsis*. In Côte d'Ivoire, recent surveys conducted by Obodji et al. (2015), N'Guessan et al. (2019) and N’guettia et al. (2021) recorded eight, nine and eleven species of mealybugs on cacao trees, respectively. Amongst these, *Pseudococcusviburni* (Signoret), identified by N’guettia et al. (2021), is the only species absent from the present report. Through the combined use of morphological identification and molecular analysis, based on DNA barcoding, eight species of mealybugs were identified from cocoa in Côte d’Ivoire for the first time, namely *D.neobrevipes*, *Pa.marginatus*, *Ph.solenopsis*, *Pl.minor*, *Ps.concavocerarii*, *Ps.occiduus*, *M.ugandae* and *Fe.dasylirii*. *Pa.marginatus*, *Ph.solenopsis* and *D.neobrevipes* are significant pests of papaya, cotton and pineapple, respectively (Germain et al. 2014, Dey et al. 2018, Rostami et al. 2024). These three species are polyphagous and can, therefore, live on various host plants (Joshi et al. 2010, Germain et al. 2014, Rostami et al. 2024). Most of the mealybug species identified in this study are amongst the 14 species known to be vectors of CSSV in cocoa (Entwistle and Longworth 1963, Roivainen 1976, Dufour 1991). However, six species, namely *D.neobrevipes*, *Pa.marginatus*, *Ph.solenopsis*, *Pl.minor*, *Ps.occiduus* and *Fe.dasylirii* have not been reported as CSSV vectors. Nevertheless, their presence in cocoa plantations could increase the risk of CSSV spread, as mealybugs are globally considered as potential plant virus vectors (Entwistle and Longworth 1963). Additionally, *Fe.dasylirii* and *Fe.virgata* are in the same genus and are morphologically similar, with morphological and molecular variations within some populations (Kaydan and Gullan 2012), suggesting that *Fe.dasylirii* could have similar CSSV transmission capabilities as *Fe.virgata*. The same applies to *D.neobrevipes*, the same genus and a species morphologically and genetically similar to *D.brevipes*, which has been identified as a vector of CSSV on cocoa (Roivainen 1976). These two *Dysmicoccus* species are recognised as good vectors of the Pineapple Mealybug Wilt-associated Virus (PMWaV), an *Ampelovirus* from the family Closteroviridae, transmitted in a semi-persistent manner, similar to the mode of transmission of CSSV (Jahn et al. 2003, Dey et al. 2018). *Pa.marginatus* is an invasive mealybug known to attack at least 54 plant families, particularly papaya, causing significant economic losses. Originally from the Americas, it has rapidly spread to Asia and Africa (Germain et al. 2014, Rostami et al. 2024). *Pa.marginatus*, along with *M.hirsutus*, are vectors of the Mulberry Mosaic Virus (MMV) in mulberries, a tospovirus from the family Bunyaviridae, transmitted persistently in a non-propagative manner (Naik et al. 2013). However, while *M.ugandae* has been confirmed as a vector of CSSV in cacao (Roivainen 1976, N’Guessan et al. 2019), there is currently no direct evidence demonstrating the ability of *M.hirsutus* to transmit this virus. Nevertheless, *M.hirsutus* may possess similar vectoring capacities to *M.ugandae*, given their morphological similarity and shared taxonomic classification within the same genus. Consequently, the presence of *Pa.marginatus* on cacao trees represents a potential risk, as this species may adapt, become a major cacao pest and possibly transmit diseases, including CSSV. *Ph.solenopsis*, known as the cotton mealybug, is a notorious polyphagous pest capable of infesting many other plants (Joshi et al. 2010, Germain et al. 2014). However, no information about its ability to transmit viruses to plants is available. Similarly, the status of *Pseudococcusocciduus* as a virus vector, particularly for CSSV, has never been demonstrated, as this study is the first to report the presence of this mealybug species on cacao trees in West Africa. The only previous mention of *Ps.occiduus* on cacao dates back to De Lotto (1964), who recorded it in Uganda. Additionally, Couturier et al. (1985) reported its presence in Côte d'Ivoire, in the dense Taï Forest in the south-western region of the country, during a mealybug survey, but not on cacao trees. Further research is needed to assess whether this species could be a vector of CSSV, as many colonies are now established in cocoa orchards in Côte d'Ivoire. In terms of occurrences, the species most frequently found in cocoa plantations in Côte d'Ivoire are *Fo.njalensis*, the *Pl.citri*/*kenyae*/*minor* group and *D.neobrevipes*. Except for *D.neobrevipes*, which is starting to become well-established in Ivorian orchards, our observations confirm those of Obodji et al. (2015), N'Guessan et al. (2019) and N’guettia et al. (2021), who reported that *Fo.njalensis* and *Pl.citri* are the dominant species in Côte d'Ivoire's cocoa orchards. These two species are also recognised as the primary vectors of several forms of CSSV in cocoa trees (Entwistle and Longworth 1963, Roivainen 1976, Dufour 1991). Their abundance in cocoa plantations significantly increases the risk of spread of the disease.

Most of the mealybug species reported in the present study were associated with ants. In total, 54 tending ant species were identified in this inventory. In Ghana, Strickland (1951b) reported around 70 ant species associated with Pseudococcidae vectors of CSSV in cocoa trees, whereas in Togo, Dufour (1991) recorded 39 ant species tending mealybugs in cocoa plantations. The tending ant community, reported in the present study, is similar to those recorded in Ghana and Togo. However, 15 additional ant species were recorded tending mealybugs in Côte d’Ivoire, namely *Ca.maculatus*, *Ca.solon*, *Ca.aff.solon*, *Cr.solenopsides*, *L.cocazela* and *M.invidium*, *M.pharaonis*, *N.angulatus*, *Para. longicornis*, *Ph.punctulata*, *Pl.intermedia*, *Ta.lugubre*, *Ta.melanocephalum* and *Tec.aff.pallipes*. Before this study, data on ants tending mealybugs on cocoa were scarce for Côte d'Ivoire. However, some information on certain species, including *Camponotus* sp., *Oe.longinoda*, *Cr.africana*, *Crematogaster* sp. and *Ph.megacephala*, can be found in the studies conducted by Alibert (1951), Risbec (1955) and Babacauh (1982). A study of ant diversity conducted by Yeo et al. (2011) near tropical forests and in cocoa plantations in the Oumé Region recorded 155 species belonging to 43 genera. Most of the species recorded in our study were listed by these authors. Association between ants and mealybugs is generally considered as an adaptive strategy for ants to easily access a regular food source, namely the honeydew produced by mealybugs (Dufour 1991). In return, tending ants protect the mealybugs from natural enemies and climatic threats like heavy rains by enclosing them in tents constructed with plant debris or soil (Dufour 1991). Cornwell (1955) also noted that the interaction between ants and mealybugs stimulates reproduction in the latter. In the present study, ant species that were found tending mealybugs the most frequently were *Ca.acvapimensis*, *Cr.africana*, *L.cocazela*, *Pa.longicornis* and *Ph.megacephala*. *Oe.longinoda*, *Od.troglodytes* and some species of *Crematogaster* seem to become increasingly dominant in their relationship with mealybugs (ADKK pers. obs.). Many other species have been occasionally found with mealybugs. Though less common, the sum of these associations represents a significant part of the mealybug-ant interactions and contribute to the complexity of the system.

The mealybug’s natural enemies identified in this study included 22 primary parasitoids, eight hyperparasitoids and 23 predators. The species community we have described in our study is richer than those reported in the past in Togo, Ghana and Côte d’Ivoire. For instance, Strickland (1951a) reported 13 parasitoid species (Encyrtidae) of *Fo.njalensis* in Ghana. More recently, Ackonor and Mordjifa (1999) and Ackonor (2002), identified seven species of parasitoids of *Fo.njalensis* and *Pl.citri* in Ghana. In Togo, Dufour (1991) also identified eight primary parasitoid species, seven secondary parasitoids and eight predators (Cecidomyiidae, Coccinellidae, Chrysopidae and Lycaenidae) of *Fo.njalensis*, *Pl.citri*/*kenyae*, *Fe.virgata* and *M.ugandae*. In Côte d’Ivoire, Risbec (1955) identified six primary parasitoid species and two hyperparasitoid species in colonies of *Fo.njalensis*. The present inventory lists nine parasitoid species of cocoa mealybugs unreported in Africa until now, namely Ac.aff.dysmicocci, *Ae.advena*, *Aloencyrtus* sp, *An.kamali*, An.aff.pseudococci, *Cocco.pulvinariae*, Cl.aff.corrugata, G.aff.tebygi and Za.aff.natalensis and one hyperparasitoid namely *Pa.muscarum*. In addition to these species, two primary parasitoids, *Bl.insularis* and *Cocci.pseudococci* and one hyperparasitoid, *Ch.cyanonotus*, have not been reported on cocoa mealybugs in Côte d’Ivoire. Some of these species like *Ae.abengouroui*, *An.kivuensis*, *An.pseudococci* and *L.dactylopii* are well-known parasitoids of mealybugs on different crops. Three of them, *An.pseudococci*, *An.kivuensis* and *Le.dactylopii*, in fact, are exotic species introduced to Ghana between 1951 and 1955 to control mealybug vectors of CSSV in cocoa plantations (Entwistle 1972). However, Risbec (1949) reported its presence in Côte d'Ivoire in the same year, stating that "this species, already described from the Belgian Congo, is undoubtedly widespread throughout tropical Africa". Similarly, Risbec (1949) also recorded *Leptomastixlongipennis* Merc., which is now recognised as a junior synonym of *L.dactylopii* Howard. This observation, therefore, predates its presumed first introduction to Ghana. Today, these species are very common in Côte d'Ivoire, where they are the primary parasitoids of *Fo.njalensis* and *Pl.citri*. Additionally, *An.kamali*, a parasitoid used in Egypt to control *M.hirsutus*, a major pest of *Hibiscus* (Moore 1988), is reported here for the first time on cocoa mealybugs in West Africa, likely parasitising these same species in cocoa plantations. Prinsloo and Annecke (1979) indicate that *Cocci.pseudococci* is an ectoparasite of *Coccodiplosiscoffeae* (Cecidomyiidae), a predator of mealybugs. However, it is worth noting that *Cocci.pseudococci* has also been observed parasitising *Fo.njalensis* and *Pl.citri* as a primary parasitoid, which is confirmed by our observations of *Cocci.pseudococci* emerging from mealybug mummies in the laboratory. In our study, *Ch.carinatus* is the dominant hyperparasitoid, which is in line with the report by Dufour (1991), who noted that *Ch.carinatus* primarily parasitised *L.bifasciata*, *Ae.abengouroui* and several species of *Anagyrus* spp. *Ch.carinatus* was also reported as the most abundant hyperparasitoid in Nigeria (Donald 1956) and Ghana (Ackonor and Mordjifa 1999). This study also highlights the presence in cocoa mealybug colonies of 11 species of predatory ladybugs, seven morpho-species of predatory gall midges, four species of predatory spiders, one species of predatory Lycaenidae and three other species of Lepidoptera, whose functional status has yet to be established. Although reliable species level identification could not be obtained in this study, our results are in line with findings of Dufour 1991 and Ackonor (2002), who indicated in their respective studies conducted in Ghana and Togo that the main predators of CSSV mealybugs were *Scymnuskibonotensis*, *Scymnus* sp., *Platynaspissolieri*, *Platynaspishingginsi*, *Hyperaspisquadrilla*, *Hyperaspisegregia*, the larvae of gall midges of the species *Coccodiplosiscoffeae* and Lepidoptera from the family Lycaenidae. Donald (1956) also reported *Nephusornatulatus* as a predator of *Ferrisiavirgata*, *Phenacoccusmadeirensis* and *Pseudococcuslongispinus* on cacao in Nigeria. Additionally, Majer (1975) recorded two other species on cacao by pyrethrum knockdown. Our study also reports the presence of large numbers of four species of ladybugs from the genus *Nephus*. Gall midges are the most frequently found predators within colonies of *Fo.njalensis* and *Pl.citri*, amongst many other mealybug species. Only the larvae are capable of preying on mealybugs at all developmental stages (Dufour 1991, Ackonor 2002).

## Conclusions

This study provides a first curated DNA barcode database to identify mealybugs and associated arthropods involved in the transmission of CSSV disease to cocoa. Through intensive sampling, we report a total of 17 mealybug species, including eight species new for cocoa in Côte d’Ivoire, a significant diversity of ants (54 species) tending mealybugs and a notable diversity of natural enemies of cocoa mealybugs, including 14 unreported species of primary parasitoids, two hyperparasitoids, 11 species of predatory ladybirds and seven species of predatory Cecidomyiidae. The fragments of COI used in this study allow for effective species identification, even between closely-related species. In all, of the 192 haplotype sequences (beyond 2% of divergence) obtained for mealybugs, ants and their natural enemies, 151 are newly provided here and made available on GenBank. This database provides valuable references for the rapid and accurate identification of entomofauna associated with CSSV disease on cocoa in Côte d’Ivoire. It provides a solid foundation for developing integrated pest management strategies based on metabarcoding in cocoa plantations and promoting a biological control approach, based on the conservation and promotion of natural biodiversity.

## Supplementary Material

B30A99C1-C877-5CD2-A764-549B8CEE100B 10.3897/BDJ.13.e144017.suppl1 Supplementary material 1 Phylogenetic tree of mealybugs Data type Phylogenetic tree of mealybugs reconstructed using COI sequences Brief description Neighbour-joining tree recontructed in PhyML 3.0 using 37 COI sequences. File: oo_1257315.pdf https://binary.pensoft.net/file/1257315 A.D.K. Koffi, R. Babin, G. Delvare, S. Chérasse, D. Ouvrard, E.M. Shimbori, K.J.H. Koigny, K.S. Kpangui, L. Benoit, M. Galan, D.C.V. Yode, S-W.M. Ouali N’Goran, J. Haran

42C802D4-3F7E-5A37-B7A3-092393D73B51 10.3897/BDJ.13.e144017.suppl2 Supplementary material 2 Detailed genetic distance table for mealybugs at species level Data type K2P genetic distances Brief description The Kimura-2-Parameter pairwise genetic distances between COI sequences for Mealybugs of CSSV obtained/used in the study. File: oo_1257293.xls https://binary.pensoft.net/file/1257293 A.D.K. Koffi, R. Babin, G. Delvare, S. Chérasse, D. Ouvrard, E.M. Shimbori, K.J.H. Koigny, K.S. Kpangui, L. Benoit, M. Galan, D.C.V. Yode, S-W.M. Ouali N’Goran, J. Haran

31FCBAB1-D195-5C20-8DC8-F8445CCED3EC 10.3897/BDJ.13.e144017.suppl3 Supplementary material 3 Phylogenetic tree of ants Data type Phylogenetic tree of ants reconstructed using COI sequences Brief description Neighbour-joining tree reconstructed in PhyML 3.0 using 76 COI sequences. File: oo_1190694.pdf https://binary.pensoft.net/file/1190694 A.D.K. Koffi, R. Babin, G. Delvare, S. Chérasse, D. Ouvrard, E.M. Shimbori, K.J.H. Koigny, K.S. Kpangui, L. Benoit, M. Galan, D.C.V. Yode, S-W.M. Ouali N’Goran, J. Haran

B8E0D846-355A-5E9E-8951-D3772C68E51B 10.3897/BDJ.13.e144017.suppl4 Supplementary material 4 Detailed genetic distance table for ants Data type K2P genetic distances Brief description The Kimura-2-Parameter pairwise genetic distances between COI sequences for ants obtained/used in the study. File: oo_1190699.xls https://binary.pensoft.net/file/1190699 A.D.K. Koffi, R. Babin, G. Delvare, S. Chérasse, D. Ouvrard, E.M. Shimbori, K.J.H. Koigny, K.S. Kpangui, L. Benoit, M. Galan, D.C.V. Yode, S-W.M. Ouali N’Goran, J. Haran

6C97B3AD-B554-5C64-9733-984D31BACFD8 10.3897/BDJ.13.e144017.suppl5 Supplementary material 5 Phylogenetic tree of parasitoids and hyperparasitoids Data type Phylogenetic tree reconstructed of parasitoids and hyperparasitoids using COI sequences Brief description Neighbour-Joining tree reconstructed in PhyML 3.0 using 39 COI sequences from 29 species. File: oo_1190714.pdf https://binary.pensoft.net/file/1190714 A.D.K. Koffi, R. Babin, G. Delvare, S. Chérasse, D. Ouvrard, E.M. Shimbori, K.J.H. Koigny, K.S. Kpangui, L. Benoit, M. Galan, D.C.V. Yode, S-W.M. Ouali N’Goran, J. Haran

32527FB8-431D-5444-B3D0-5958F2BB43D4 10.3897/BDJ.13.e144017.suppl6 Supplementary material 6 Detailed genetic distance table for parasitoids and hyperparasitoids Data type K2P genetic distances Brief description The Kimura-2-Parameter pairwise genetic distances between COI sequences for parasitoids and hyperparasitoids obtained/used in the study. File: oo_1190715.xls https://binary.pensoft.net/file/1190715 A.D.K. Koffi, R. Babin, G. Delvare, S. Chérasse, D. Ouvrard, E.M. Shimbori, K.J.H. Koigny, K.S. Kpangui, L. Benoit, M. Galan, D.C.V. Yode, S-W.M. Ouali N’Goran, J. Haran

E76528AD-FB63-55B6-A9ED-57EC40E5FC88 10.3897/BDJ.13.e144017.suppl7 Supplementary material 7 Phylogenetic tree of Coccinellidae predators Data type Phylogenetic tree of Coccinellidae reconstructed using COI sequences Brief description Neighbour-Joining tree reconstructed in PhyML 3.0, using 15 COI sequences for 11 species. File: oo_1190740.pdf https://binary.pensoft.net/file/1190740 A.D.K. Koffi, R. Babin, G. Delvare, S. Chérasse, D. Ouvrard, E.M. Shimbori, K.J.H. Koigny, K.S. Kpangui, L. Benoit, M. Galan, D.C.V. Yode, S-W.M. Ouali N’Goran, J. Haran

96A64F70-B08D-508A-9AD0-1F5F13A1D055 10.3897/BDJ.13.e144017.suppl8 Supplementary material 8 Detailed genetic distance table for Coccinellidae predator Data type K2P genetic distances Brief description The Kimura-2-Parameter pairwise genetic distances between COI sequences for Coccinellidae predators obtained/used in the study. File: oo_1190744.xls https://binary.pensoft.net/file/1190744 A.D.K. Koffi, R. Babin, G. Delvare, S. Chérasse, D. Ouvrard, E.M. Shimbori, K.J.H. Koigny, K.S. Kpangui, L. Benoit, M. Galan, D.C.V. Yode, S-W.M. Ouali N’Goran, J. Haran

6B3A1132-F110-5074-8550-348362816D76 10.3897/BDJ.13.e144017.suppl9 Supplementary material 9 Phylogenetic tree of Ceccidomyiidae predators Data type Phylogenetic tree of Ceccidomyiidae predators reconstructed using COI sequences Brief description Neighbour-Joining tree reconstructed in PhyML 3.0, using nine COI sequences for seven morpho-species of Ceccidomyiidae. File: oo_1190749.pdf https://binary.pensoft.net/file/1190749 A.D.K. Koffi, R. Babin, G. Delvare, S. Chérasse, D. Ouvrard, E.M. Shimbori, K.J.H. Koigny, K.S. Kpangui, L. Benoit, M. Galan, D.C.V. Yode, S-W.M. Ouali N’Goran, J. Haran

58DC7128-AD1E-5DED-8E5C-6437EC473C15 10.3897/BDJ.13.e144017.suppl10 Supplementary material 10 Detailed genetic distance table of Ceccidomyiidae morpho-species Data type K2P genetic distances Brief description The Kimura-2-Parameter pairwise genetic distances between COI sequences for Ceccidomyiidae morpho-species obtained/used in the study. File: oo_1190748.xls https://binary.pensoft.net/file/1190748 A.D.K. Koffi, R. Babin, G. Delvare, S. Chérasse, D. Ouvrard, E.M. Shimbori, K.J.H. Koigny, K.S. Kpangui, L. Benoit, M. Galan, D.C.V. Yode, S-W.M. Ouali N’Goran, J. Haran

ED8C2820-96ED-54F0-B06B-1352C16C629E 10.3897/BDJ.13.e144017.suppl11 Supplementary material 11 Phylogenetic tree of other natural enemies Data type Phylogenetic tree reconstructed using COI sequences Brief description Neighbour-Joining tree reconstructed in PhyML 3.0, using 14 COI sequences from four species of spiders, one species of Lepidoptera and eight species of parasitoids of the predators Ceccidomyiidae, Lepidoptera and Coccinellidae. File: oo_1190820.pdf https://binary.pensoft.net/file/1190820 A.D.K. Koffi, R. Babin, G. Delvare, S. Chérasse, D. Ouvrard, E.M. Shimbori, K.J.H. Koigny, K.S. Kpangui, L. Benoit, M. Galan, D.C.V. Yode, S-W.M. Ouali N’Goran, J. Haran

10DE1DE3-C0C4-595E-BD34-641C17319522 10.3897/BDJ.13.e144017.suppl12 Supplementary material 12 Detailed genetic distance table for other natural enemies Data type K2P genetic distances Brief description The Kimura-2-Parameter pairwise genetic distances between COI sequences for other natural enemies obtained/used in the study. File: oo_1190826.xls https://binary.pensoft.net/file/1190826 A.D.K. Koffi, R. Babin, G. Delvare, S. Chérasse, D. Ouvrard, E.M. Shimbori, K.J.H. Koigny, K.S. Kpangui, L. Benoit, M. Galan, D.C.V. Yode, S-W.M. Ouali N’Goran, J. Haran

4E671C77-CE0E-5967-BEC4-D644612B9B15 10.3897/BDJ.13.e144017.suppl13 Supplementary material 13 FASTA file of Mealybugs COI sequences Data type Genetic sequences of mealybugs Coding for 385 bp Brief description FASTA file containing the sequences of different haplotypes of the 19 mealybug species with their VOUCHER codes."GenBank accession numbers will be made available pending acceptance of the manuscript". File: oo_1216921.txt https://binary.pensoft.net/file/1216921 A.D.K. Koffi, R. Babin, G. Delvare, S. Chérasse, D. Ouvrard, E.M. Shimbori, K.J.H. Koigny, K.S. Kpangui, L. Benoit, M. Galan, D.C.V. Yode, S-W.M. Ouali N’Goran, J. Haran

74578774-0C76-57A0-9BC7-301BC688BCA7 10.3897/BDJ.13.e144017.suppl14 Supplementary material 14 FASTA file of ants COI sequences Data type Genetic COI sequences of ants coding for 418 bp Brief description FASTA file containing the sequences of different haplotypes of the 54 ant species with their VOUCHER codes. "GenBank accession numbers will be made available pending acceptance of the manuscript". File: oo_1191324.txt https://binary.pensoft.net/file/1191324 A.D.K. Koffi, R. Babin, G. Delvare, S. Chérasse, D. Ouvrard, E.M. Shimbori, K.J.H. Koigny, K.S. Kpangui, L. Benoit, M. Galan, D.C.V. Yode, S-W.M. Ouali N’Goran, J. Haran

70D62D3B-8AC9-5902-89B1-773E745C2EFE 10.3897/BDJ.13.e144017.suppl15 Supplementary material 15 FASTA file of parasitoids and hyperparasitoids COI sequences Data type Genetic COI sequences of parasitoids and hyperparasitoids coding for 418 bp Brief description FASTA file containing the sequences of different haplotypes of the 29 parasitoids and hyperparasitoids species with their VOUCHER codes. "GenBank accession numbers will be made available pending acceptance of the manuscript". File: oo_1191325.txt https://binary.pensoft.net/file/1191325 A.D.K. Koffi, R. Babin, G. Delvare, S. Chérasse, D. Ouvrard, E.M. Shimbori, K.J.H. Koigny, K.S. Kpangui, L. Benoit, M. Galan, D.C.V. Yode, S-W.M. Ouali N’Goran, J. Haran

D6D6F32F-09B2-52E5-A854-AE743F19F237 10.3897/BDJ.13.e144017.suppl16 Supplementary material 16 FASTA file of Coccinellidae COI sequences Data type Genetic COI sequences of Coccinellidae predators coding for 418 bp Brief description FASTA file containing the sequences of different haplotypes of the 11 species of Coccinellidae with their VOUCHER codes. "GenBank accession numbers will be made available pending acceptance of the manuscript". File: oo_1191326.txt https://binary.pensoft.net/file/1191326 A.D.K. Koffi, R. Babin, G. Delvare, S. Chérasse, D. Ouvrard, E.M. Shimbori, K.J.H. Koigny, K.S. Kpangui, L. Benoit, M. Galan, D.C.V. Yode, S-W.M. Ouali N’Goran, J. Haran

84440EEE-36E3-588A-BECC-880816E2AF66 10.3897/BDJ.13.e144017.suppl17 Supplementary material 17 FASTA file of Ceccidomyiidae COI sequences Data type Genetic COI sequences of Ceccidomyiidae coding for 418 bp Brief description FASTA file containing the sequences of different haplotypes of the seven morpho-species of Ceccidomyiidae with their VOUCHER codes. "GenBank accession numbers will be made available pending acceptance of the manuscript". File: oo_1191320.txt https://binary.pensoft.net/file/1191320 A.D.K. Koffi, R. Babin, G. Delvare, S. Chérasse, D. Ouvrard, E.M. Shimbori, K.J.H. Koigny, K.S. Kpangui, L. Benoit, M. Galan, D.C.V. Yode, S-W.M. Ouali N’Goran, J. Haran

C04C0CB5-2503-54CC-94D8-A80FB780EBEC 10.3897/BDJ.13.e144017.suppl18 Supplementary material 18 FASTA file of other natural enemies COI sequences Data type Genetic COI sequences of other natural enemies coding for 418 bp Brief description FASTA file containing the sequences of different haplotypes of the 13 additional predators of mealybugs and parasitoids of mealybug predators, along with their VOUCHER codes. "GenBank accession numbers will be made available pending acceptance of the manuscript". File: oo_1191329.txt https://binary.pensoft.net/file/1191329 A.D.K. Koffi, R. Babin, G. Delvare, S. Chérasse, D. Ouvrard, E.M. Shimbori, K.J.H. Koigny, K.S. Kpangui, L. Benoit, M. Galan, D.C.V. Yode, S-W.M. Ouali N’Goran, J. Haran

## Figures and Tables

**Table 1.** Location of sampling sites.

Sample site name	Longitude (X)	Latitude (Y)	Mealybug and associated ant sampling	Natural enemy sampling
Aboisso	-3.32031	5.47451	x	x
Azaguié	-4.48282	5.57077	x	x
Soubré	-6.49383	5.74143	x	
Guiglo	-7.87404	6.53766	x	x
Man	-7.24257	7.30263	x	x
Bonon	-6.04698	6.91921	x	x
Adzopé	-3.72629	5.97444	x	x
Grand-Bereby	-7.00418	4.84994	x	x
Agnibilekro	-3.20366	7.12785	x	
Guibéroua	-6.21471	6.21354	x	x
Meagui	-6.81134	5.59470	x	x
San Pedro	-6.46408	4.89055	x	x
Guéyo	-6.16756	5.80025	x	
Blé	-5.17393	5.95151	x	
Fresco	-5.57361	5.13914	x	x

**Table 2.** Inventory of cocoa-feeding mealybugs (Hemiptera, Pseudococcidae) sampled in Côte d’Ivoire, with an indication of their frequency: (-) a rare occurrence, (+) moderate occurrence, (++) regular occurrence and (+++) very frequent occurrence.

**Species**	**Author**	**Institutional codes for uniques haplotypes**	**GenBank accession codes**	**Number of** **specimens sequenced**	**Maximal genetic distance (%)**	**Frequency in Field Observation and collection**
*Dysmicoccusbrevipes*	(Cockerell, 1893)	FAUN17932	PV050941	7	0	++
*Dysmicoccusneobrevipes*	Beardsley, 1959	FAUN17895	PV050942	44	0	+++
*Ferrisiavirgata*	(Cockerell, 1893)	FAUN17882	PV050943	7	0	+
*Ferrisiadasylirii*	(Cockerell, 1896)	FAUN17883	PV050944	4	0	+
*Formicococcusnjalensis*	(Laing, 1929)	FAUN18614 FAUN17871 FAUN18412 FAUN17747 FAUN17866 FAUN17943 FAUN17811	PV050931 PV050932 PV050933 PV050934 PV050935 PV050936 PV050937	154	4.57	+++
*Maconellicoccushirsutus*	(Green, 1908)	FAUN17794	PV050945	9	0	++
*Maconellicoccusugandae*	(Laing, 1925)	FAUN17930	PV050946	1		-
*Paracoccusmarginatus*	Williams & Granara de Willink, 1992	FAUN18502	PV050947	2	0	-
*Phenacoccushargreavesi*	(Laing, 1925)	FAUN17919 FAUN17939 FAUN17937	PV050938 PV050939 PV050940	8	2.38	++
*Phenacoccussolenopsis*	Tinsley, 1898	FAUN18314	PV050948	1		-
*Planococcuscitri*	(Risso, 1813)	FAUN17856 FAUN17767 FAUN17857 FAUN17722 FAUN17987	PV050923 PV050924 PV050925 PV050926 PV050927	24	2.93	+++
*Planococcuskenyae*	(Le Pelley, 1935)	FAUN17845	PV050928	9	0	++
*Planococcusminor*	(Maskell, 1897)	FAUN17808 FAUN18242	PV050929 PV050930	9	1.84	+
*Pseudococcusconcavocerarii*	James, 1934	FAUN17933 FAUN17881	PV050949 PV050950	10	1.05	++
*Pseudococcusjackbeardsleyi*	Gimpel & Miller, 1996	FAUN17907	PV050951	9	0	++
*Pseudococcuslongispinus*	(Targioni Tozzetti, 1867)	FAUN17887 FAUN17890 FAUN17909	PV050952 PV050953 PV050954	3	3.73	+
*Pseudococcusocciduus*	De Lotto, 1961	FAUN17926 FAUN17903 FAUN17911	PV050955 PV050956 PV050957	4	6.57	+

**Table 3.** Inventory of ants (Hymenoptera, Formicidae) tending cocoa mealybugs in Côte d'Ivoire, with indication on their frequency: (-) a rare occurrence, (+) moderate occurrence, (++) regular occurrence and (+++) very frequent occurrence.

Species	Author	Institutional codes for uniques haplotypes	GenBank accession codes	Number of specimens sequenced	Maximal genetic distance (%)	Frequency in Field Observation and collection
*Atopomyrmexmocquerysi*	André, 1889	FAUN17842	PV051035	3	0.48	-
*Camponotusacvapimensis*	Mayr, 1862	FAUN17952 FAUN18517 FAUN17893	PV051036 PV051037 PV051038	11	1.45	+++
*Camponotusmaculatus*	(Fabricius, 1782)	FAUN17715	PV051039	1		-
*Camponotusaff.solon*	Forel, 1886	FAUN17716	PV051040	2	0	-
*Camponotussolon*	Forel, 1886	FAUN17717	PV051041	2	0	-
*Camponotus* sp.1		FAUN17989 FAUN17709	PV051042 PV051043	10	0.72	++
*Camponotus* sp.2		FAUN17972	PV051044	1		-
*Camponotus* sp.3		FAUN17976	PV051045	1		-
*Camponotus* sp.4		FAUN18519	PV051046	1		-
*Cataulacusguineensis*	Smith,1853	FAUN18337	PV051047	1		-
*Crematogasterafricana*	Mayr, 1895	FAUN17718 FAUN17719 FAUN17720 FAUN18510	PV051048 PV051049 PV051050 PV051051	7	1.21	+++
*Crematogasterclariventris*	Mayr, 1895	FAUN17760	PV051052	1		-
*Crematogastersolenopsides*	Emery, 1899	FAUN18551	PV051053	1		+
*Crematogasterstadelmanni*	Mayr, 1895	FAUN17722	PV051054	5	0	++
*Crematogaster* sp.1		FAUN17738 FAUN17743 FAUN18514	PV051055 PV051056 PV051057	11	2.69	++
*Crematogaster* sp.2		FAUN17733 FAUN18507	PV051058 PV051059	3	0.72	+
*Crematogaster* sp.3		FAUN17747 FAUN17761	PV051060 PV051061	4	1.21	++
*Crematogaster* sp.4		FAUN17943 FAUN17757	PV051062 PV051063	3	0.48	+
*Crematogaster* sp.5		FAUN18563	PV051064	3	0	+
*Crematogaster* sp.6		FAUN17756	PV051065	1		-
*Crematogaster* sp.7		FAUN18560	PV051066	1		-
*Crematogaster* sp.8		FAUN17721	PV051067	1		-
*Crematogaster* sp.9		FAUN17755	PV051068	1		-
*Crematogaster* sp.10		FAUN17736	PV051069	9	0	++
*Lepisiotacocazela*	Santschi, 1926	FAUN17791	PV051070	10	0	+++
*Lepisiota* sp.		FAUN17792	PV051071	1		-
*Monomoriumfloricola*	(Jerdon, 1851)	FAUN17805	PV051072	2	0	+
*Monomoriuminvidium*	Bolton, 1987	FAUN17809 FAUN17815	PV051074 PV051075	4	0	+
*Monomoriumpharaonis*	(Linnaeus, 1758)	FAUN17811	PV051073	4	0	-
*Monomorium* sp.		FAUN17806	PV051076	1		-
*Nylanderia* sp.1		FAUN17813	PV051077	2	0	-
*Nylanderia* sp.2		FAUN17820	PV051078	1		-
*Odontomachustroglodytes*	Santschi, 1914	FAUN17783	PV051079	10	0	+
*Oecophyllalonginoda*	(Latreille, 1802)	FAUN17823 FAUN17824	PV051080 PV051081	3	1.21	++
*Nesomyrmexangulatus*	(Mayr, 1862)	FAUN18543	PV051082	1		-
*Paratrechinalongicornis*	(Latreille, 1802)	FAUN17818 FAUN17804	PV051083 PV051084	4	6.29	++
*Pheidolecrassinoda*	Emery, 1895	FAUN17774	PV051085	5	0	+
*Pheidolemegacephala*	(Fabricius, 1793)	FAUN17965 FAUN17766	PV051086 PV051087	9	2.70	+++
*Pheidolepunctulata*	Mayr, 1866	FAUN17781 FAUN18562	PV051088 PV051089	3	4.98	-
*Pheidole* sp.1		FAUN17780 FAUN17765 FAUN17958 FAUN17770 FAUN17775 FAUN17740	PV051090 PV051091 PV051092 PV051093 PV051094 PV051095	42	3.96	+++
*Pheidole* sp.2		FAUN17773 FAUN17779	PV051096 PV051097	2	1.70	+
*Pheidole* sp.3		FAUN18498	PV051098	2	0	++
*Pheidole* sp.4		FAUN17769	PV051099	1		+
*Plagiolepisintermedia*	Emery, 1895	FAUN17821	PV051100	1		+
*Plagiolepis* sp.		FAUN17814	PV051101	2	0	-
*Solenopsis* sp.		FAUN18532	PV051102	1		-
*Strumigenysconcolor*	Santschi, 1914	FAUN18545	PV051103	1		-
*Tapinomalugubre*	Santschi, 1917	FAUN17936	PV051104	6	0	+
*Tapinomamelanocephalum*	(Fabricius, 1793)	FAUN18527	PV051105	1		-
Technomyrmexaff.pallipes	Mayr, 1872	FAUN17801	PV051106	3	0	-
*Tetramorium* sp.1		FAUN17843 FAUN17844	PV051107 PV051108	2	0.72	+
*Tetramorium* sp.2		FAUN17838	PV051109	1		-
*Tetramorium* sp.3		FAUN17840	PV051110	1		-
*Tetramorium* sp.4		FAUN17841	PV051111	1		-

**Table 4.** Inventory of natural enemies (parasitoid Hymenoptera, Diptera, Coleoptera, Lepidoptera and Araneae) of cocoa mealybugs in Côte d’Ivoire, with indication on their frequency: (-) rare occurrence, (+) moderate occurrence, (++) regular occurrence and (+++) very frequent occurrence. The ten last species (marked with *; **; ***) are respectively the parasitoids of Lepidopteran (Lepidoptera), Cecidomyiidae (Diptera) and Coccinellidae (Coleoptera)

**Species**	**Author**	**Functional status**	**Institutional codes for uniques haplotypes**	**GenBank accession codes**	**Number of** **specimens sequenced**	**Maximal genetic distance (%)**	**Frequency in Field Observation and collection**
Acerophagusaff.dysmicocci	(Bennett, 1955)	Parasitoid	FAUN18136	PV050958	1		-
*Acerophagus* sp.		Parasitoid	FAUN18134 FAUN18135	PV050959 PV050960	2	0.49	-
*Aenasiusabengouroui*	(Risbec, 1949)	Parasitoid	FAUN18087 FAUN18100 FAUN18093	PV050961 PV050962 PV050963	10	0.98	+++
*Aenasiusadvena*	Compere, 1937	Parasitoid	FAUN18112	PV050964	1		+
*Aloencyrtus* sp.	Prinsloo,1978	Parasitoid	FAUN18147	PV050965	1		-
*Anagyrusamoenus*	Compere, 1939	Parasitoid	FAUN18074a	PV050966	2	0	-
*Anagyruskamali*	Moursi, 1948	Parasitoid	FAUN18076	PV050967	2	0	++
*Anagyruskivuensis*	Compere, 1939	Parasitoid	FAUN18062	PV050968	6	0	+++
Anagyrusaff.pseudococci	(Girault, 1915)	Parasitoid	FAUN18068	PV050969	1		++
Anagyrusaff.subproximus	(Silvestri, 1915)	Parasitoid	FAUN18058 FAUN18059	PV050970 PV050971	2	8.22	+
*Blepyrusinsularis*	Cameron, 1886	Parasitoid	FAUN18085	PV050972	2	0	+
*Chartocerus* sp.		Hyperparasitoid	FAUN18142	PV050973	2		-
*Cheiloneuruscarinatus*	(Compere, 1938)	Hyperparasitoid	FAUN18033 FAUN18051 FAUN18057	PV050974 PV050975 PV050976	10	1.47	+++
Cheiloneurusaff.carinatus	(Compere, 1938)	Hyperparasitoid	FAUN18053	PV050977	1		+
*Cheiloneuruscyanonotus*	Waterston, 1917	Hyperparasitoid	FAUN18045a	PV050978	1		-
*Coccidoctonuspseudococci*	(Risbec, 1954)	Parasitoid	FAUN18021 FAUN18118	PV050979 PV050980	11	0	+++
*Coccidoxenoïdes* sp.		Hyperparasitoid	FAUN18144		1		-
*Coccophaguspulvinariae*	Compere, 1931	Parasitoid	FAUN18143	PV050981	1		-
*Clauseniacorrugata*	Kerrich, 1967	Parasitoid	FAUN18083	PV050982	1		+
Clauseniaaff.corrugata	Kerrich, 1967	Parasitoid	FAUN18081a	PV050983	1		+
*Clausenia* sp.		Parasitoid	FAUN18084	PV050984	1		-
Gyranusoideaaff.tebygi	Noyer, 1988	Parasitoid	FAUN18077	PV050985	1		-
*Gyranusoidea* sp.		Parasitoid	FAUN18074b		1		-
*Leptomastixdactylopii*	Howard, 1885	Parasitoid	FAUN18000 FAUN18012 FAUN18015 FAUN18116	PV050986 PV050987 PV050988 PV050989	10	5.85	+++
*Pachyneuronmuscarum*	(Linnaeus, 1758)	Hyperparasitoid	FAUN18146	PV050990	1		-
*Prochiloneurusaegyptiacus*	(Mercet, 1929)	Hyperparasitoid	FAUN18046	PV050991	1		-
*Promuscidea* sp.	(Ghesquière, 1955)	Hyperparasitoid	FAUN18131	PV050992	3	0	+
Zaplatycerusaff.natalensis	Compere, 1939	Parasitoid	FAUN18121 FAUN18123	PV050993 PV050994	5	0	++
*Zaplatycerusafricanus*	Compere, 1939	Parasitoid	FAUN18124	PV050995	1		++
*Zaplatycerus* sp.		Parasitoid	FAUN18113	PV050996	1		+
*Hyperaspis* sp.		Predator	FAUN18202 FAUN18203	PV050997 PV050998	2	2.44	+
*Nephus* sp.1		Predator	FAUN18196 FAUN18205	PV050999 PV051000	7	1.45	++
*Nephus* sp.2		Predator	FAUN18206	PV051001	1		+
*Nephus* sp.3		Predator	FAUN18198	PV051002	1		+
*Nephus* sp.4		Predator	FAUN18195	PV051003	1		++
*Platynaspis* sp.1		Predator	FAUN18188	PV051004	1		+
*Platynaspis* sp.2		Predator	FAUN18187	PV051005	1		-
*Platynaspis* sp.3		Predator	FAUN18194	PV051006	1		-
*Scymnus* sp.1		Predator	FAUN18189 FAUN18190	PV051007 PV051008	2	1.21	++
*Scymnus* sp.2		Predator	FAUN18200	PV051009	2	0	++
Scymnus sp.3		Predator	FAUN18191 FAUN18192	PV051010 PV051011	2	1.70	+
Cecidomyiidae sp.1		Predator	FAUN18183	PV051012	3	0	+++
Cecidomyiidae sp.2		Predator	FAUN18165 FAUN18161	PV051013 PV051014	7	2.44	+++
Cecidomyiidae sp.3		Predator	FAUN18167 FAUN18160	PV051015 PV051016	2	2.20	++
Cecidomyiidae sp.4		Predator	FAUN18166	PV051017	2	0	++
Cecidomyiidae sp.5		Predator	FAUN18174	PV051018	1		+
Cecidomyiidae sp.6		Predator	FAUN18186	PV051019	1		+
Cecidomyiidae sp.7		Predator	FAUN18168	PV051020	1		+
*Myrmarachnesp*.		Predator	FAUN18150	PV051030	1		-
*Niditinea* sp.		-	FAUN18210		2	0	-
*Psammitis* sp.		Predator	FAUN18149	PV051033	1		-
*Spalgislemolea*		Predator	FAUN18212	PV051034	1		-
*Syringoseca* sp.		-	FAUN18213		1		-
*Theridion* sp.1		Predator	FAUN18153	PV051031	1		-
*Theridion* sp.2		Predator	FAUN18154	PV051032	1		-
*Antrocephalus* sp*		Parasitoid	FAUN18117	PV051029	1		-
*Apanteles* sp.*		Parasitoid	FAUN18145	PV051028	1		-
Aphanogmusaff.fumipennis**	Thomson, 1859	Parasitoid	FAUN18125	PV051024	1		+
*Aphanogmus* sp.1**		Parasitoid	FAUN18127	PV051025	1		-
*Aphanogmus* sp.2**		Parasitoid	FAUN18129	PV051026	1		-
Homalotylusaff.oculatus***	(Ratzeburg,1844)	Parasitoid	FAUN18018 FAUN18042	PV051022 PV051021	4	2.48	-
Homalotylusaff.africanus***	Timberlake, 1919	Parasitoid	FAUN18043	PV051023	1		-
*Ooencyrtus* sp.*		Parasitoid	FAUN18081b		1		-
*Platygaster* sp.**		Parasitoid	FAUN18128	PV051027	1		-
