# Supplementary figures and images for: A barcode database for insects associated with the spread of the Cocoa Swollen Shoot Virus Disease in Côte d’Ivoire
Source: Biodivers Data J. 2025 Mar 14;13:e144017. doi: 10.3897/BDJ.13.e144017 (PMC11929007; doi:10.3897/BDJ.13.e144017)

0.10 0.20 0.30 0.40 0.50 0.60 0.70

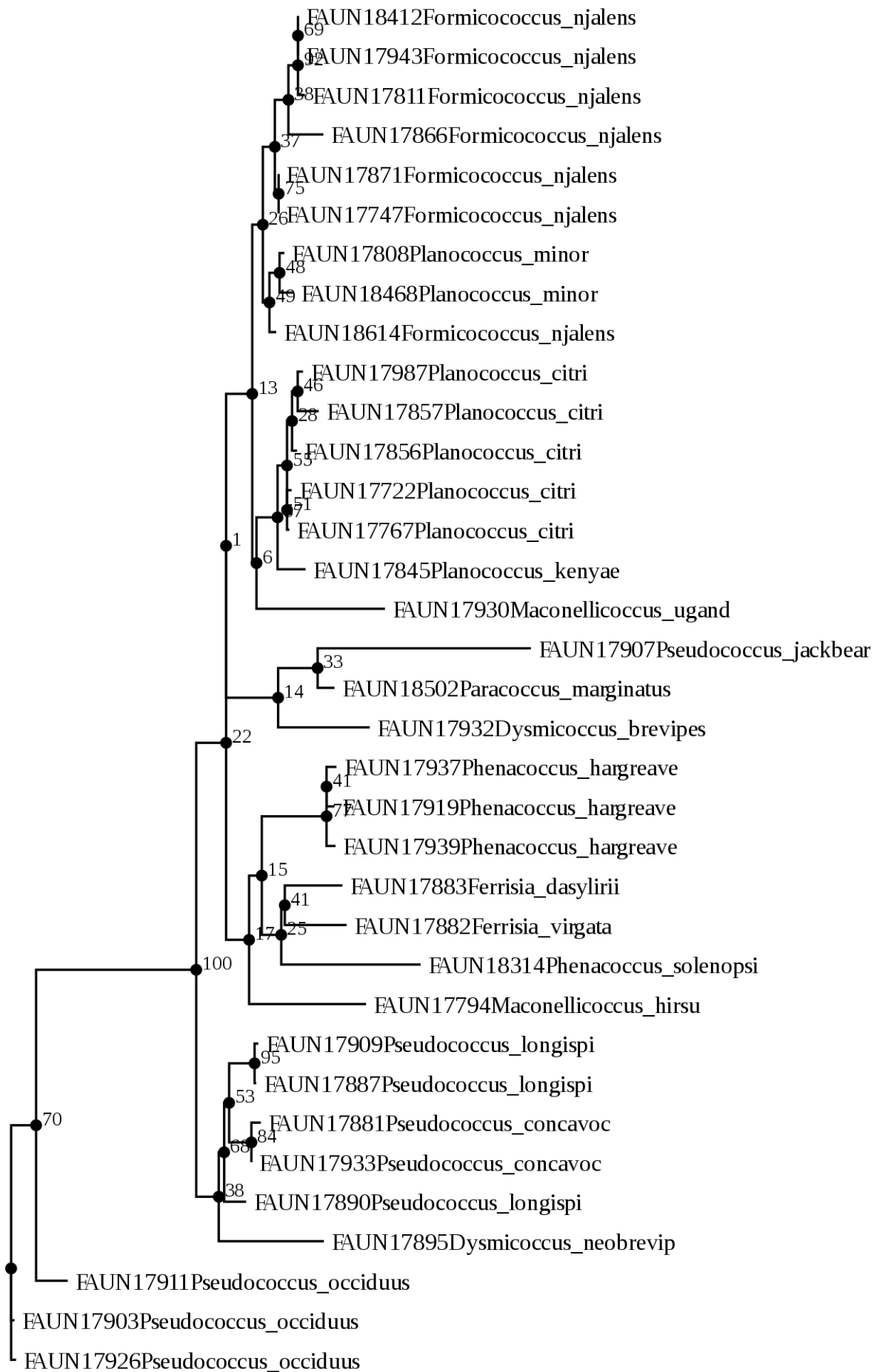

Supplement: Supplementary material 1 — Phylogenetic tree of mealybugs [file bdj-13-e144017-s001.pdf]

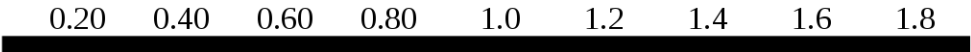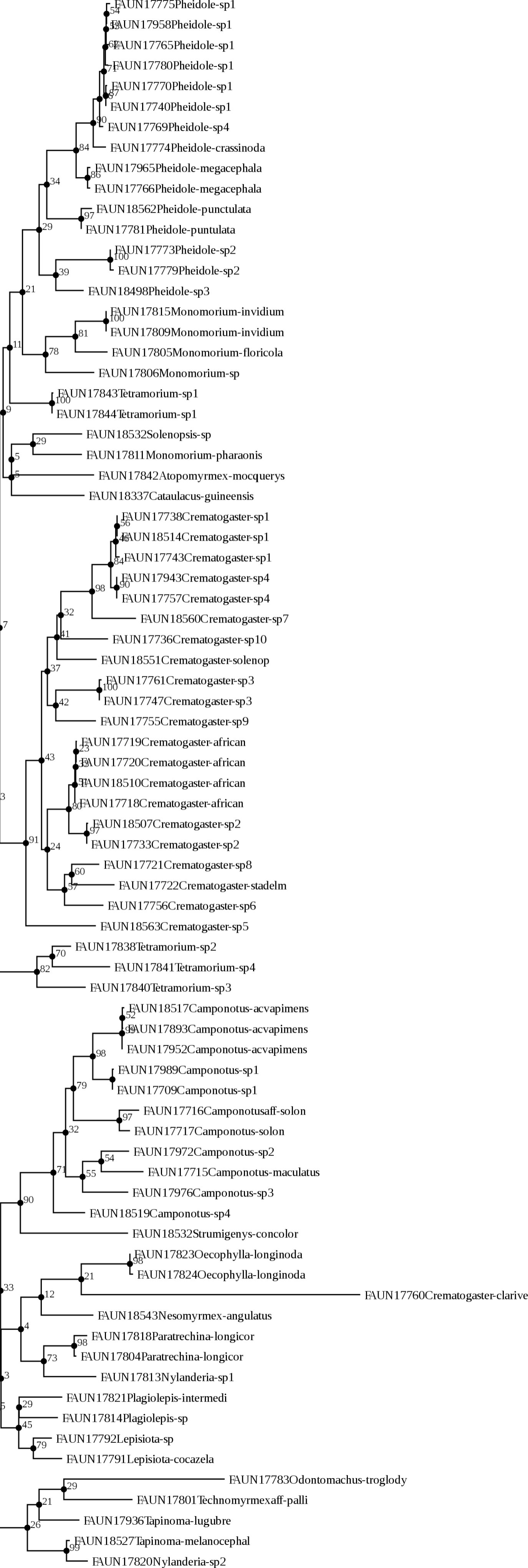

Supplement: Supplementary material 3 — Phylogenetic tree of ants [file bdj-13-e144017-s003.pdf]

0.10 0.20 0.30 0.40 0.50 0.60 0.70 0.80

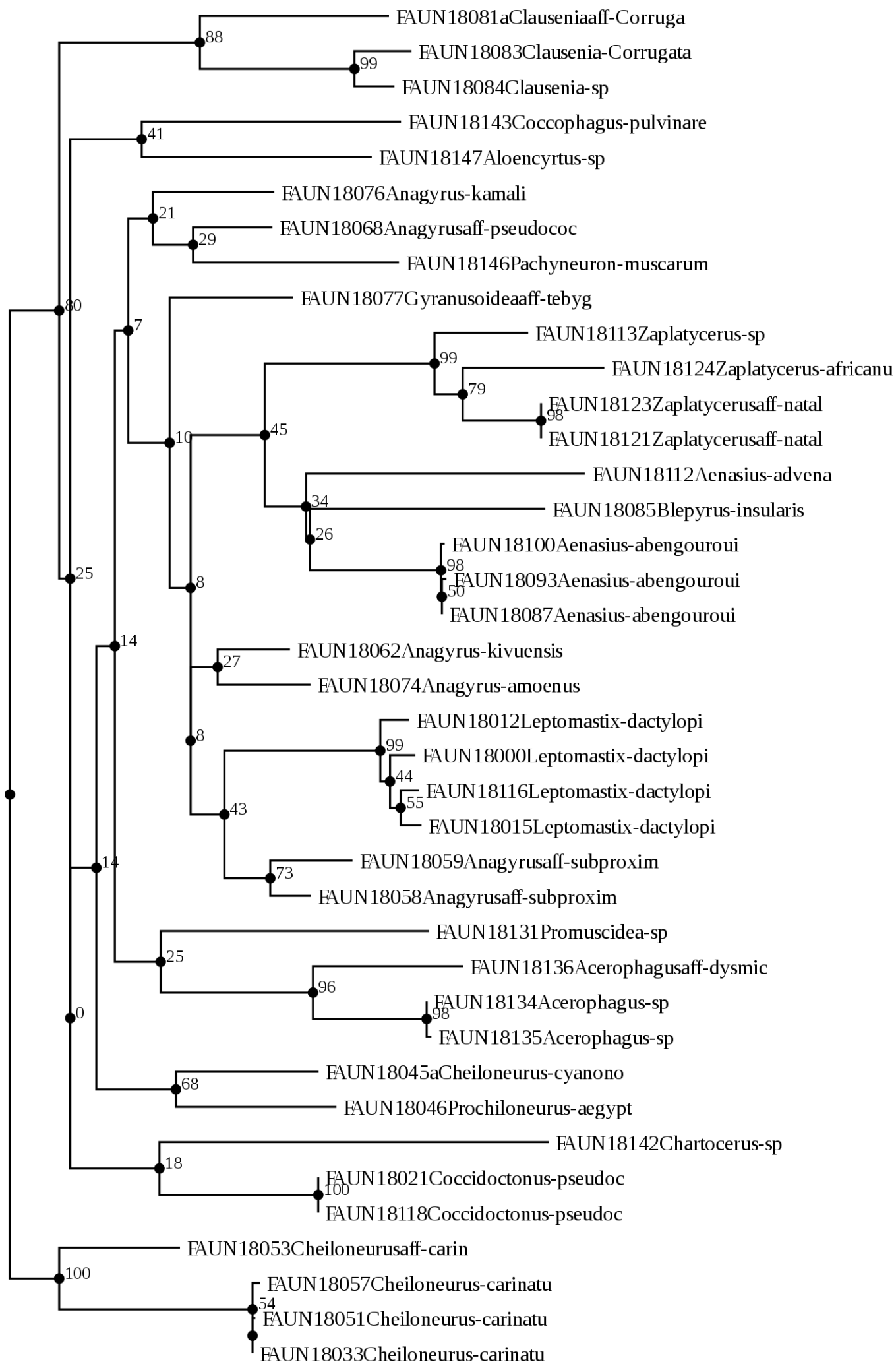

Supplement: Supplementary material 5 — Phylogenetic tree of parasitoids and hyperparasitoids [file bdj-13-e144017-s005.pdf]

0.10 0.20 0.30

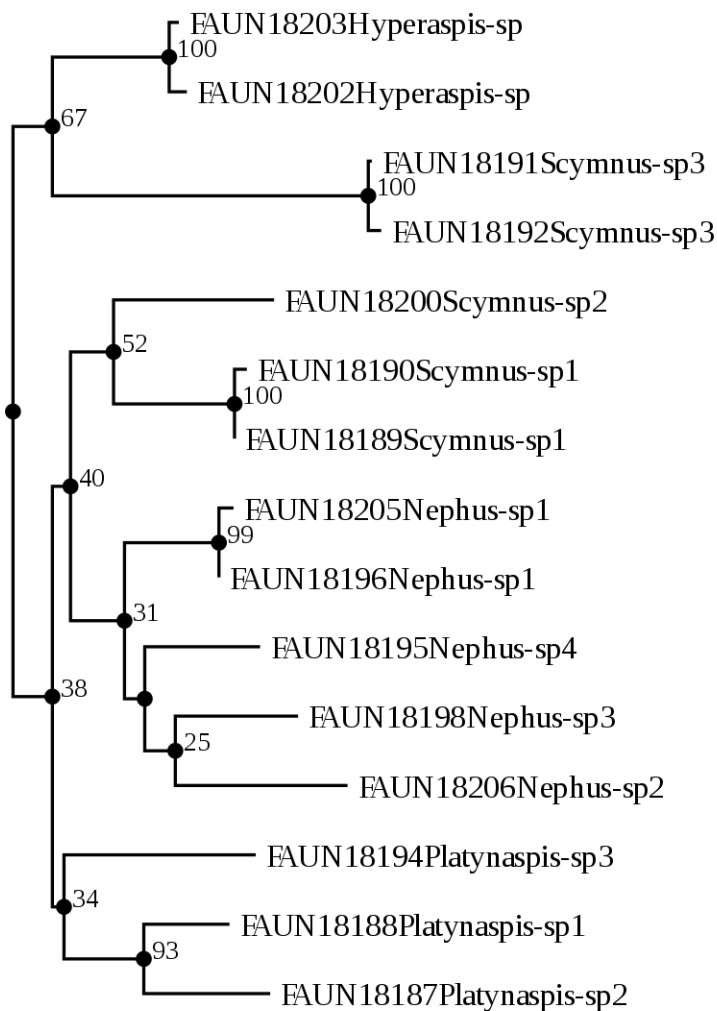

Supplement: Supplementary material 7 — Phylogenetic tree of Coccinellidae predators [file bdj-13-e144017-s007.pdf]

0.20      0.40

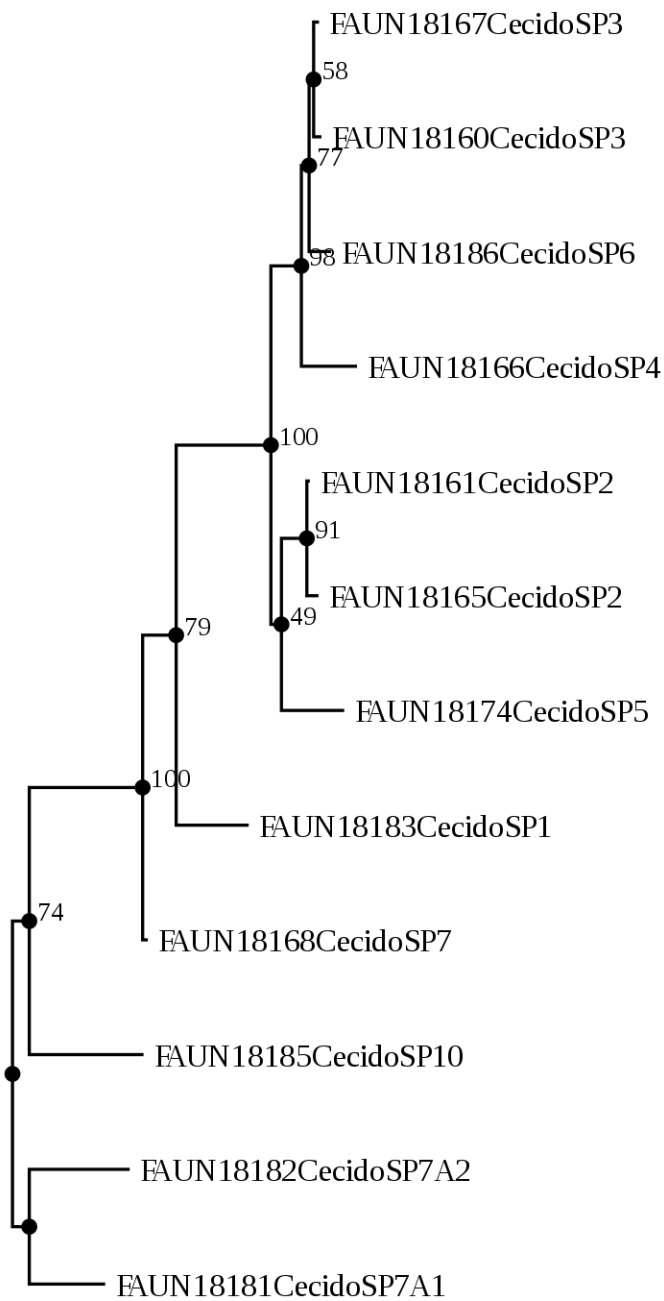

Supplement: Supplementary material 9 — Phylogenetic tree of Ceccidomyiidae predators [file bdj-13-e144017-s009.pdf]

0.10 0.20 0.30 0.40 0.50 0.60 0.70

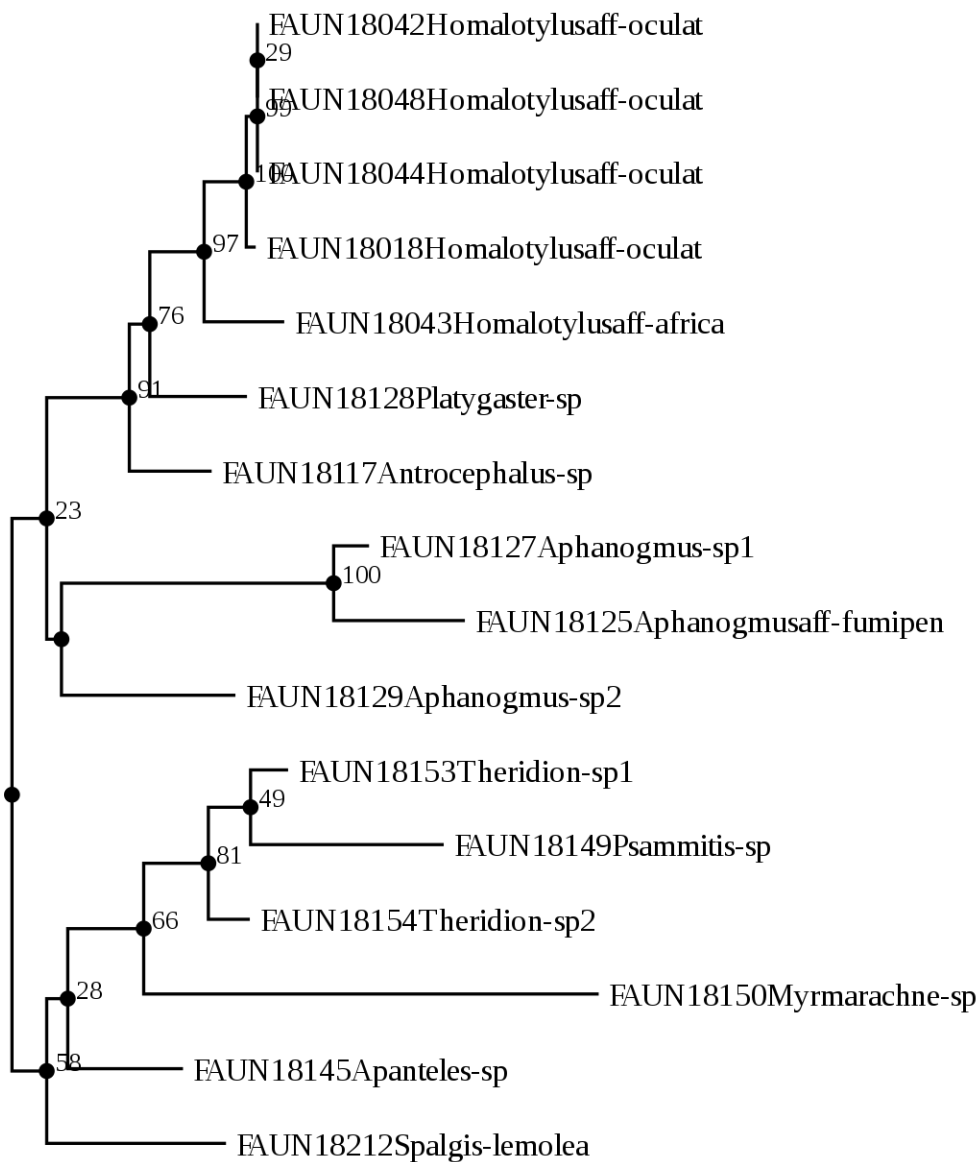

Supplement: Supplementary material 11 — Phylogenetic tree of other natural enemies [file bdj-13-e144017-s011.pdf]
